# Understanding Non-Pharmacological Treatments for Fibromyalgia Functional and Well-Being Status: The Role of Literacy

**DOI:** 10.3390/healthcare12191956

**Published:** 2024-09-30

**Authors:** Anca Maria Amzolini, Carmen Daniela Neagoe, Taina Elena Avramescu, Adina Mitrea, Rodica Traistaru, Elena Simona Micu, Simona Laura Ianoşi, Daniela Matei

**Affiliations:** 1 Department Medical Semiology, University of Medicine and Pharmacy Craiova, 200349 Craiova, Romania; anca.amzolini@umfcv.ro (A.M.A.); simona.micu@umfcv.ro (E.S.M.); 2 Department of Internal Medicine, University of Medicine and Pharmacy Craiova, 200349 Craiova, Romania; daniela.neagoe@umfcv.ro; 3 Sport Medicine and Physiotherapy, University of Craiova, 200585 Craiova, Romania; taina_mistico@yahoo.com; 4 Department of Diabetes, Nutrition and Metabolic Diseases, University of Medicine and Pharmacy Craiova, 200349 Craiova, Romania; 5 Department of Medical Rehabilitation, University of Medicine and Pharmacy Craiova, 200349 Craiova, Romania; rodicatraistaru@umfcv.ro (R.T.); mateidana30@yahoo.com (D.M.); 6 Department of Dermatolgy, University of Medicine and Pharmacy Craiova, 200349 Craiova, Romania; simona.ianosi@umfcv.ro

**Keywords:** fibromyalgia, literacy, level of education, functional status

## Abstract

Background/Objectives: Fibromyalgia (FM) affects up to 5% of the global population and is a leading cause of significant social and economic consequences. Higher health literacy leads to better understanding of treatment plans, improved self-care, and adherence to recommendations, enhancing overall quality of life. This study aims to determine whether different aspects of the disease are influenced by patients’ education level and literacy when applying the same therapy and to assess how patients’ perceptions of therapy outcomes vary over time based on their educational level. Methods: This study involved 140 fibromyalgia (FM) patients diagnosed using the 2016 ACR criteria, with 128 completing the study. Participants attended three visits over 28 weeks and were stratified into four groups based on educational level: Group 1—secondary school or less; Group 2—high school graduates; Group 3—college graduates; Group 4—university graduates. Patients were assigned to groups (n = 32, 32, 30, and 34, respectively) after the initial evaluation (T0). The treatment was assessed (T1) and followed up three months later (T2) to evaluate changes in functional status and quality of life. All patients underwent the same rehabilitation program, cognitive therapy, and kinesiotherapy. Results: Significant differences in disease impact on the patient’s life (FIQ total score) were observed between groups from the initial evaluation (*p* = 0.000). The overall FIQ score was notably affected by non-pharmacological therapy in patients with higher education. These differences continued to be significant even three months after the treatment ended (*p* = 0.000). Functional limitations were evident from the start (*p* = 0.000) and improved significantly post-treatment in patients with higher education (*p* = 0.000). However, subjective evaluations of disease impact (assessed by the first item of FIQ) did not consistently align with objective findings (hand grip strength). Functional limitations did not significantly differ in subjective evaluations (F1Q1) across educational levels (*p* = 0.045), and inverse correlations were noted between functional status and SF-12 well-being components. Conclusions: This study underscores that higher education enhances fibromyalgia management and functional outcomes, particularly when combined with non-pharmacological therapies. However, subjective perceptions may not always align with objective improvements, indicating that factors beyond education, such as personal and external influences, also impact disease management. Thus, improving health literacy through educational interventions could further benefit FM patients’ quality of life.

## 1. Introduction

Fibromyalgia (FM) affects up to 5% of the global population [1] and is a leading cause of chronic pain among adults, resulting in significant social and economic consequences [2]. The condition, characterized by widespread pain, stiffness, fatigue, sleep disorders, and cognitive issues, predominantly affects middle-aged women [3]. These symptoms, often accompanied by anxiety and depression, greatly impact daily life [4]. An internet survey of over 2500 respondents with FM found that forgetfulness and concentration problems were common complaints, following pain, fatigue, muscle tension, and sleep disturbances [5].

Despite its prevalence, the characteristics and existence of FM remain controversial, relying heavily on clinical diagnosis [6]. Some physicians question the legitimacy of fibromyalgia syndrome and worry that diagnosing it may lead to overuse of healthcare resources [7], as the diagnosis heavily relies on the examiner’s clinical judgment. Despite these concerns, substantial evidence supports the symptoms reported by fibromyalgia patients. Recent research suggests a new model for understanding the disease’s pathogenesis, incorporating elements of a pain-processing thalamocortical loop [8] and introducing the concept of nociplastic pain. It is believed that peripheral immune–endocrine signaling, combined with genetic factors and cognitive–emotional mechanisms, contributes to neuroplastic changes in pain processing pathways [9]. However, studies suggest that costs decrease once a diagnosis is made [10], as patients no longer need to see multiple specialists. Various diagnostic criteria have been proposed, with the American College of Rheumatology (ACR) introducing updates in 2010, 2016, and 2019 to address the limitations of earlier criteria [11,12]. The ACR also developed a survey questionnaire in 2011 to facilitate large-scale epidemiological and clinical studies [2].

Recent literature reviews highlight sex-related differences in FM, especially given its higher prevalence among women [13]. Studies indicate that socioeconomic status, age, and individual personality traits also influence pain thresholds [1,14,15,16]. Individuals with chronic conditions, like fibromyalgia, are in a uniquely challenging situation [17]. They must find a balance between adhering to a treatment plan and maintaining their daily activities as normally as possible. These patients need to develop skills for managing their symptoms and the overall impact of their condition, which requires constant and active involvement in their care [18]. Sociocultural and economic factors are more evident contributors to differences in health status, potentially affecting how symptoms are perceived and the effectiveness of treatment [19]. Although it remains unclear whether fibromyalgia symptom severity is directly linked to socioeconomic status, factors such as poor mental health may partially explain this relationship [20]. Additionally, social support plays a crucial role in fibromyalgia management, given the psychological aspects of the condition, including feelings of negativity, isolation, and helplessness [21]. Franks’ study emphasized the significant importance of both the quantity and quality of social support in treatment outcomes [22].

Numerous therapeutic trials for FM have produced uncertain results, with patient compliance often limiting the duration and significance of these studies. In the United States, three medications—pregabalin, duloxetine, and milnacipran—are approved for FM treatment; however, in Romania, none of these drugs are approved for the treatment of patients with FM [23,24].

Non-pharmacological therapies, such as physical therapy and cognitive-behavioral therapy (CBT), are also important. Physical therapy combines methods and techniques to cure, prevent, and rehabilitate patients with various impairments [25]. CBT, based on the cognitive theory of emotional responses, aims to modify dysfunctional thought patterns and behaviors to improve patient outcomes [26]. Massage is a common therapeutic approach for managing key symptoms of fibromyalgia (FM), offering not only pain relief but also improvements in sleep quality, depression, and anxiety [27]. Acupuncture and electroacupuncture, widely used for pain management in FM, are popular non-pharmacological treatments. However, research has shown that their analgesic effects tend to be modest [28]. Additionally, emerging evidence highlights the positive effects of physical exercise, hyperbaric oxygen therapy, and non-invasive brain stimulation for FM, though these treatments need further validation through comprehensive studies [8]. Fibromyalgia is increasingly viewed as a combination of physical, psychological, and social impairments, following the bio–psycho–social model [29]. This approach integrates pharmacological treatment with physical, psychological, behavioral, and educational interventions. Studies that systematically compare single therapies to combined treatments—including pharmacologic, physical, and psychosocial elements—consistently show that combination therapies lead to better outcomes for chronic pain [30]. The most effective approach for treating FM appears to be a combination of medical treatment (including analgesics and antidepressants), physical therapy, and psychotherapy (such as relaxation techniques and cognitive-behavioral therapy) [31].

Research underscores the importance of patient adherence to medication regimens for successful FM treatment. Physicians must ensure patients understand their medication plans, potential side effects, and the importance of consistent use, with adherence strongly influenced by health literacy [32]. Effective collaboration is also crucial for successful kinesiotherapy and CBT, with good communication, mutual respect, and active patient involvement being key [33,34].

A recent systematic review examined the health literacy of patients experiencing musculoskeletal pain, including fibromyalgia. The review concluded that it is still uncertain how much and in what way low health literacy levels impact the effectiveness of supported self-management for musculoskeletal conditions [35].

Given the diversity of symptoms and therapeutic options, evaluating effectiveness of treatment strategies in FM patients is challenging [36,37,38]. This study aims to determine whether different aspects of the disease are influenced by patients’ education level and literacy when applying the same therapy and to assess how patients’ perceptions of therapy outcomes vary over time based on their educational level.

## 2. Materials and Methods

### 2.1. Design Overview

We conducted an interventional prospective comparative cohort study that took place from May 2022 to March 2024 at the Internal Medicine and Rehabilitation Clinics of two affiliated hospitals of the University of Medicine and Pharmacy in Craiova, Romania.

The patients were stratified based on their education levels as follows: Group 1—secondary school or less; Group 2—high school graduates; Group 3—college graduates (postsecondary education or technical qualification); Group 4—university graduates.

After the initial evaluation (T0), the study patients were assigned to one of four groups, according to the patient’s educational level: Group 1 (n = 32), Group 2 (n = 32), Group 3 (n = 30), or Group 4 (n = 34). The first evaluation of the treatment efficacy was assessed at 4 weeks from the beginning of the non-pharmacological applied treatment (T1). To evaluate subjective and objective changes in terms of functional status and quality of life, a follow-up assessment was conducted at 3 months after treatment (T2).

### 2.2. Study Group

The study group consisted of 140 patients diagnosed with FM (2019 ACR criteria), with 128 subjects completing the study (only patients who completed and accurately participated in all evaluations and attended at least 70% of the program sessions were included in the statistical analysis) and providing valid data. Eligible patients were informed about the study and provided with the necessary informed consent documentation. The patients signed the informed consent knowingly, after being informed about the purpose of the study, the advantages and disadvantages of participating, potential side effects, details regarding the confidentiality of personal data, and the fact that they could withdraw from the study at any time without providing further explanation, and without facing any consequences. The population sample included four groups of patients previously diagnosed with FM. The specifics of each group are as follows:

- Group 1: Initially, 35 FM patients with a secondary education level or less showed interest in participating in the interventional program. Ultimately, 32 patients were selected for the database (meeting the criteria for full participation in all evaluations and attending at least 70% of the program sessions). This translates to a participation rate of 91.4% of the initially interested patients.
- Group 2: All 35 FM patients with college-level education who were initially selected accepted participation in the program. However, only 32 participants were included in the database (meeting the criteria for full participation in all evaluations), resulting in a 91.4% participation rate among the initially interested patients.
- Group 3: This group initially included 35 patients, but only 30 of them completed the program and were considered for analysis, resulting in an 85.7% completion rate.
- Group 4: Of the 35 patients who initially accepted participation, 34 were included in the database. This translates to a 97.1% participation rate among the initially interested patients.

Inclusion Criteria were: age over 18 years; the patients signed the patient’s informed consent form; confirmed previous diagnosis of FM according to the 2019 ACR criteria; patients monitored by a physician; stable pharmacological treatment for at least 3 months prior; ability to speak and understand the language; regardless of the type of treatment administered for disease control; patients were allowed to use analgesic/anti-inflammatory medications, as they had before the study (the patients did not receive new drug classes during the study and the drugs dosages were not increased during this time).

Exclusion Criteria: another potential primary cause of patient’s disability that may influence the study results; inclusion in a kinetic and/or CBT program within the last 6 months; alcoholism; psychiatric disorders; any medical condition that may interfere with the symptoms of fibromyalgia (degenerative or inflammatory rheumatological conditions, orthopedic conditions); patients deemed unable (in the investigator’s opinion) to answer the study questionnaires; participation in other study.

### 2.3. Study Treatment

The non-pharmacological treatment applied was administered to patients in groups of up to 10 individuals, with each session lasting 1 h and 30 min (1 h for CBT and 30 min for kinesiotherapy).

Regarding the kinesiotherapy, patients attended three sessions per week at the gym and were encouraged to perform exercises at home two additional times per week, if their physical condition allowed. Among the forms of kinesiotherapy, stretching was selected, with the following objectives: to reduce muscle tension and promote relaxation and well-being; improve coordination by facilitating movements; increase mobility; prepare the body for activities; maintain and enhance flexibility; and improve body awareness by focusing on individual muscles. Each session commenced with a warm-up routine and encompassed exercises targeting the core of the body, as well as the upper and lower limbs. Additionally, to enhance relaxation, the exercises were accompanied by soothing music.

The CBT focused on cognitive-behavioral techniques using relaxation methods, breathing exercises, and visualization. Its objectives included identifying challenges affecting daily roles (with the patient as the focus), enhancing attention, memory, and concentration, and recognizing symptoms, situations, and responses through understanding positive and negative stress processes.

Each session was conducted and supervised by both a rehabilitation doctor and at least one kinesiology specialist. The therapy sessions followed the same guidelines for CBT and used the same type of exercises for kinesiotherapy. Additionally, the team designated to coordinate the non-pharmacological program evaluated the patients at all the scheduled time points.

### 2.4. Ethical Considerations

The most important factors for this investigation were the safety and well-being of the patients. Consequently, the investigation was conducted according to the ethical and deontological principles of the Helsinki Declaration of human rights. All the patients signed an information and acceptance form, i.e., an informed consent form, to be included in the present study. Moreover, the protocol was approved by the ethics committee of the University of Medicine and Pharmacy of Craiova, Romania (no. 80/13 May 2022).

### 2.5. Collecting Information Methods

This study considers all variables found in the medical records. We used four methods for collecting information: clinical history, interview, clinical evaluation, and self-report questionnaires. The included variables were divided into two major groups: social and demographic parameters and diagnosis and clinical parameters.

The socio-demographic parameters were assessed using the interview, including general data: age, gender, marital status, working/disability status, residing area, and level of education. Relevant behavioral data were also collected: number of cigarettes smoked, coffee intake, alcohol and drug consumption (abuse), birth control pills, use of mobile phone, social media access.

In terms of diagnosis parameters, based on the patients’ clinical history, we obtained data regarding the year of diagnosis (years of illness), the year when the pain symptoms appeared (years of pain), additional medication intake for pain control (analgesic/NSAI; no. per week), and associated conditions.

Clinical parameters included the clinical variables gathered, evaluating the most affected area in FM:

- Observed functional limitation: The reliability of hand grip strength measurements in FM patients has been demonstrated to be excellent [39]. To assess maximal voluntary hand grip force, we used an electronic EH101 hand dynamometer (precision: 0.1 kg, tolerance: 0.5 kg). Prior to data collection, the physician provided detailed instructions. Following recommendations, the handle diameter was adjusted to 19.7% of the participant’s hand length. Participants were instructed with the verbal command: “Squeeze! Harder and harder! Relax!” [40].

Grip strength was quantified on a scale of 1 to 4:

- 4 for values between 10 and 20 kg,
- 3 for values between 21 and 30 kg,
- 2 for values between 31 and 40 kg,
- 1 for values above 41 kg.

During the measurement, subjects held the dynamometer with their arm beside their trunk, shoulder in a neutral position, elbow flexed at 90°, and pulled the bar with their fingers [40]. This test was conducted on the dominant hand. The average of three trials for each side, with 2 min rest intervals to prevent fatigue, was calculated. A higher score indicated a more significant observed functional limitation.

Perceived functional limitation and disease impact on the patient’s life: The FIQ (Fibromyalgia Impact Questionnaire) consists of 10 items assessing the patient’s status over the past week [41]. The first item (FIQ1—functional impairment) includes 10 questions about how frequently the patient was able to perform household chores and daily activities such as shopping, vacuuming, cooking, making beds, washing dishes, visiting friends, and driving. Responses were rated from 0 (always) to 4 (never). The second and third items asked how many days in the last week the patient felt well and how many days they were unable to work due to FM, respectively. The remaining seven items required the patient to rate, on a scale from 1 (absent) to 10 (very intense), their levels of pain, fatigue, stiffness, anxiety, and depression, as well as how tired they felt in the morning and how FM symptoms affected their ability to work.

The final FIQ score was calculated using a specific formula, yielding results from 0 (not affected by FM) to 100 (severely affected by FM).

- Quality of life: The Short-Form Health Survey (SF-12) is a multipurpose questionnaire composed of 12 questions derived from the SF-36 Health Survey [42]. It is well established that there is an inverse correlation between the functional status of FM patients and their SF-12 scores, covering both physical and mental components [43]. The SF-12 assesses eight dimensions: physical function (2 items), social function (1 item), physical role (2 items), emotional role (2 items), mental health (2 items), vitality (1 item), bodily pain (1 item), and general health (1 item). Responses are rated on Likert scales measuring either intensity or frequency. The number of response options varies from 3 to 6, depending on the item: ranging from 1 (excellent) to 5 (poor), from 1 (yes, limited a lot) to 3 (no, not limited at all), or from 1 (all the time) to 6 (none of the time). For items 4 to 7, the answers are simply “yes” (scored 1) or “no” (scored 2).

The responses were combined, scored, and weighted to generate two scales (physical and mental health indicators) that provide insights into the quality of life related to physical and mental health, as well as overall health [44]. The scores are standardized so that a score of 50 represents the average norm. For statistical analysis, we used the scores from these two subscales.

- Medication intake: Regarding the medication intake for pain control, we evaluated the following aspects: name, usage duration, dosage, and frequency. For the statistical analysis, we used the total number of pills ingested by the patient as number per week.

### 2.6. Statistical Analysis

Version 20 of the Statistical Package for Social Sciences (SPSS) program (IBM Corporation, Armonk, NY, USA) was used to create and analyze the database. Before proceeding with data analysis, we conducted tests to assess normality and homoscedasticity, given that each sample exceeded 30 subjects. Normality was assessed using the Kolmogorov–Smirnov test, where the null hypothesis assumes normal distribution and is rejected if the significance level is ≤0.05. Most variables met the normality assumption; for those that did not, we considered the standard error of kurtosis. Despite not meeting the normality criteria, none of the variables exceeded thresholds that would necessitate non-parametric tests.

Descriptive analyses, including means and percentages, were performed to characterize the samples. ANOVA and Chi-Square tests were used to examine differences in continuous and categorical variables across the four groups. Within-group differential analyses evaluated changes over time in the variables. Paired-sample *t*-tests (repeated measures) were conducted within each group to identify significant differences between three assessment points. Mean comparisons across study time points were conducted within each sample group. Independent-sample *t*-tests and ANOVA (with Huynh–Feldt correction when sphericity assumptions were violated) assessed differences between groups.

Correlation analyses and regressions examined relationships between variables. Pearson’s two-tailed bivariate test assessed simultaneous trends in two parameters, with interpretation based on the strength of the correlation coefficient [45]. For direct correlations, results were visually represented using scatterplots and boxplots. Statistical significance was interpreted based on predefined thresholds, indicating differences in variables between treatment options, evaluation times, or correlation trends [46].

## 3. Results

Out of a total of 140 patients diagnosed with FM who agreed to participate in the study, 128 completed it (with over 85% being women). As shown in Table 1, various social and demographic parameters were quantified for the four study groups. The mean age of patients across the four study groups was comparable, with no statistically significant differences (*p* = 0.620). Regarding the place of origin, Group 4 included a significant percentage of urban patients, with a statistically significant difference compared to Groups 1, 2, and 3 (*p* = 0.009). Marital status showed a statistically significant difference between the group with higher education (university graduates, Group 4) and Group 1 for the divorced status (*p* < 0.001). While all patients had access to social media (*p* = 0.450), Group 1 included the highest percentage of disabled patients (*p* = 0.005), and Group 4 had the highest percentage of professionally active patients (*p* = 0.005). There were no differences in smoking, coffee consumption, or alcohol/drug abuse among the four study groups.

In terms of clinical parameters, we considered the disease duration prior to the inclusion of patients in the study, the duration of the most significant symptom in fibromyalgia, pain, as well as associated conditions commonly linked to the diagnosis of FM. The conditions most commonly associated with personal history in all studied groups were irritable bowel syndrome, chronic fatigue syndrome, and tension headaches. Additionally, the most frequent symptom observed in both groups was diffuse pain lasting for more than 8 years. As shown in Table 2, there were no clinical differences between groups.

Regarding the observed functional impairment, significant differences were found among the four groups at T0 (*p* < 0.001). Specifically, statistically significant difference was observed between Group 2 and patients from Group 3 (*p* < 0.001), as well as between the mean values of Group 1 and Group 3 (*p* = 0.001). Regarding the FIQ1 scores, no statistically significant differences were found between Groups 3 and 4 (*p* = 0.08), although the overall *p*-value across all four groups was statistically significant (*p* = 0.045). Significant statistical differences were evident between Group 4 and Group 1 (*p* = 0.04), and between Group 4 and Group 2 (*p* = 0.02).

At T0, significant inter-group differences were observed in the FIQ total score: between Group 4 and Group 2 (*p* < 0.001), Group 4 and Group 3 (*p* = 0.001), as well as Group 4 and Group 1 (*p* = 0.009). Highly significant differentiation was noted among the four groups regarding FIQ total values (*p* < 0.001).

The physical condition scale of the SF-12 showed statistically significant differences among all groups (*p* = 0.018), particularly between Group 4 and the other three groups: compared to Group 1 (*p* = 0.041), Group 2 (*p* = 0.012), and Group 3 (*p* = 0.004). Another significant difference noted at the initial assessment was between Group 1 and Group 3 (*p* = 0.04).

For the mental condition scale of the SF-12, significant differences were observed among the four groups at the initial evaluation (*p* = 0.001), with the most significant *p*-value observed between Group 4 and Group 1 (*p* = 0.001), followed by Group 3 (*p* = 0.004).

Regarding medication intake, significant differences were found between Group 4 and Group 2 (*p* = 0.001), between Group 4 and Group 1 (*p* = 0.001), between Group 3 and Group 2 (*p* = 0.001), and between Group 3 and Group 1 (*p* < 0.000).

### 3.1. Evolution over Time and Inter-Group Differences of the Study Parameters

#### 3.1.1. Observed Functional Limitations

Time evolution: Group 4 registered a significant decrease (*p* < 0.000) from T0 (mean: 13.7 ± 3.9) to T1 (mean: 10.3 ± 2.7) followed by an increase at T2 (mean: 12.4 ± 3.5) which holds no statistical significance when compared to the first two evaluations. Group 3 also registered a significant decrease (*p* = 0.000) between the scores at T0 (mean: 15.4 ± 2.6) and T1 (mean: 13.0 ± 3.3) evaluations, decrease (*p* = 0.000) that was still observed at T2 (mean: 13.8 ± 3.1). There were no important changes in the evolution of the other two groups.

**Figure 1:**
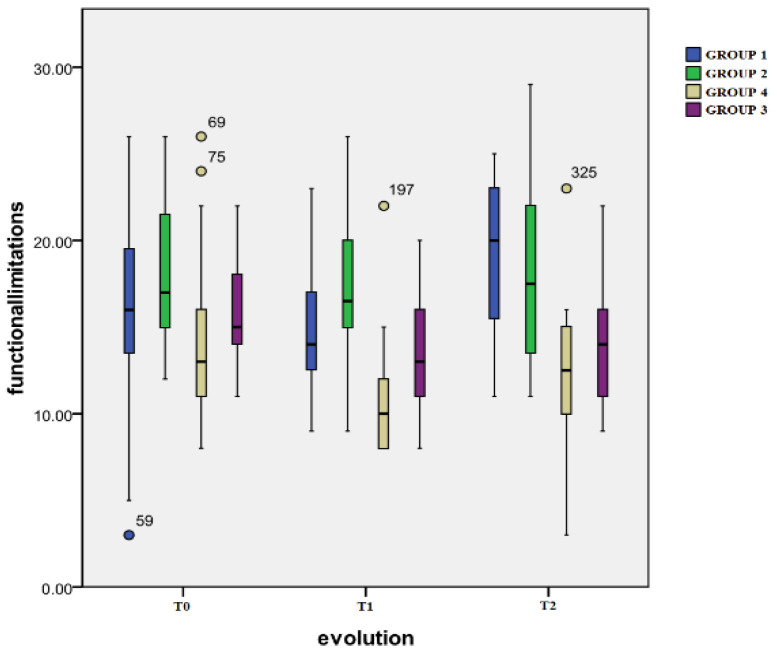
Observed functional impairment.

The results of the scores from the observed functional limitations evaluation are presented in Figure 1.

As for the inter-group differences: At T1, the differences in the scores obtained from Group 4 and the other three groups were highly significant: Group 1 (*p* = 0.000), Group 2 (*p* = 0.000), and Group 3 (*p* = 0.001). When comparing Group 3 to Group 2, the difference was also highly significant (*p* = 0.000). At follow-up evaluation (T2), Group 4 displayed significantly lower scores than Group 1 (*p* = 0.001) or Group 2 (*p* = 0.000). For Group 3, highly significant differences were also observed when compared to Group 1 (*p* = 0.000) or Group 2 (*p* = 0.000).

#### 3.1.2. Perceived Impairment (FIQ1 Scale)

Where the evolution in time is concerned, Group 4 registered the most significant differences. The initial score (mean: 12.1 ± 5.3) dropped significantly (*p* < 0.001) at T1 (mean: 9.8 ± 5.3) and then increased at T2 (mean: 10.2 ± 5.1), the difference between the first and third evaluation remained very significant (*p* = 0.006). For Group 3, the initial score (mean: 12.7 ± 4.6) decreased (*p* = 0.001) at T1 (mean: 8.6 ± 7) and T2 (mean: 9.7 ± 5.8) evaluations (*p* = 0.002).

Regarding the inter-group differences, the only significant one was registered at T2 between Group 3 and Group 1 (*p* = 0.007).

**Figure 2:**
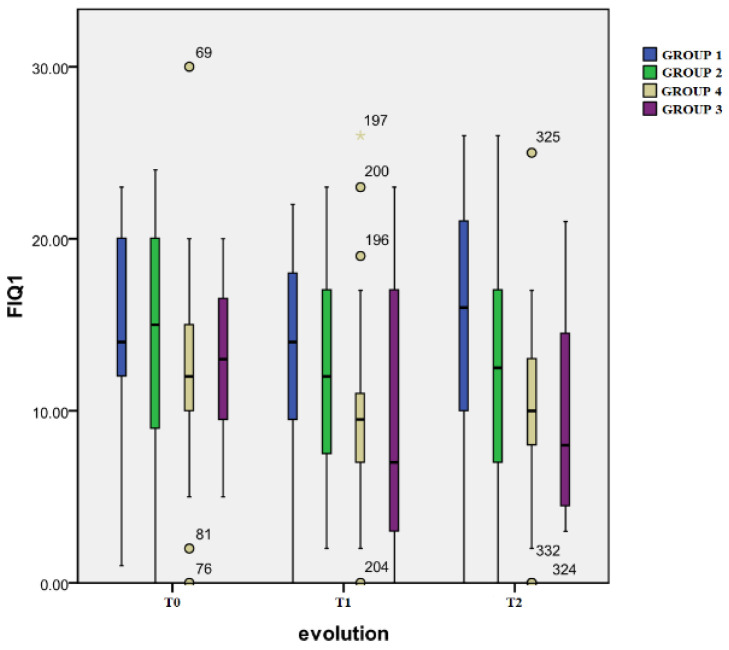
Perceived functional impairment.

The results from the functional impairment as rated by the patients on the FIQ1 scale are presented in Figure 2.

#### 3.1.3. Disease Impact on the Patient’s Life (FIQ Total)

Time evolution: For Group 1, the only significant difference was between the T0 (mean: 71.0 ± 15.9) and T1 evaluations (mean: 62.6 ± 20.2) (*p* = 0.04). The scores from Group 4 registered a significant decrease from the initial evaluation (T0) (mean: 59.3 ± 18.9) to the T1 moment (mean: 43.4 ± 18.6) (*p* < 0.001) and T2 (mean: 45.1 ± 17.9) (*p* < 0.001) moment. Group 3 also registered a highly significant change in the initial (T0) (mean: 72.5 ± 10.5) and second (T1) scores (mean: 51.0 ± 18.1) (*p* < 0.001), and the T0 and T2 (mean: 55.6 ± 17.3) scores (*p* < 0.001). And finally, for Group 2, there was a significant difference noted between the T0 (mean: 74.3 ± 11.5) and T2 evaluations (mean: 68.9 ± 17.5) (*p* = 0.016).

**Figure 3:**
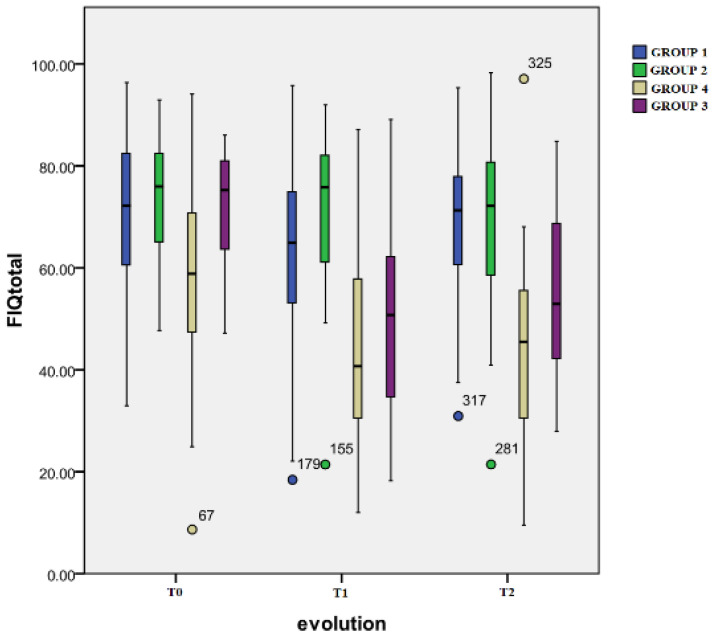
FIQ total score.

Figure 3 presents the evolution in time of the FIQ total scores for all four samples.

Inter-group differences: At T1, significant differences were observed between Group 2 and Group 4 (*p* < 0.001) or Group 3 (*p* < 0.001) and between Group 1 and Group 4 (*p* < 0.001) or Group 3 (*p* = 0.03). At T2, significant differences were registered between Group 4 and the other groups: Group 1 (*p* < 0.001), Group 2 (*p* < 0.001), and Group 3 (*p* = 0.033). Moreover, the score for Group 3 was significantly lower than the one of Group 1 (*p* = 0.009) or Group 2 (*p* = 0.008).

#### 3.1.4. Quality of Life (SF-12)

**Figure 4:**
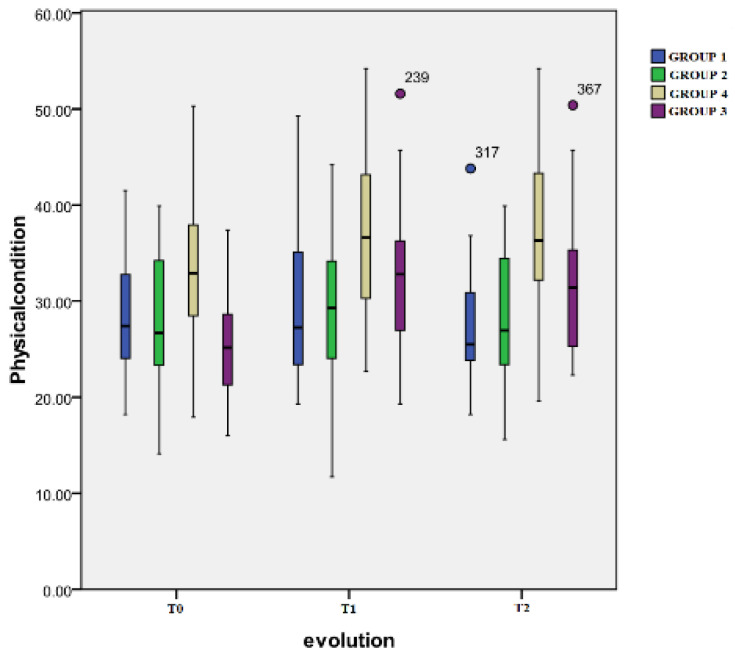
Physical health scale.

The results obtained by computing the scores from the physical condition scale of the Short Form-12 health survey are presented in Figure 4.

Regarding the evolution in time between the three evaluation moments, no significant differences were found for Group 1 or for Group 2 (*p* > 0.05). Group 3 displayed a highly significant difference between T0 (mean: 24.2 ± 13.3) and T1 (34.5 ± 12.8) (*p* < 0.001) and between T0 and T2 (mean: 33.2 ± 12.4) (*p* = 0.001). For Group 4, significant changes were seen between T0 (mean: 33.0 ± 10.1) and T1 (mean: 37.9 ± 11.3) (*p* < 0.001) and between T0 and T2 (mean: 36.8 ± 11.6) (*p* = 0.014).

Inter-group differences: At T1, the only significant differences were the ones observed between Group 4 and the other three groups: Group 1 (*p* = 0.001), Group 2 (*p* < 0.001), and Group 3 (*p* = 0.01). At T2, important differences were noted between the same Group 4 and the other ones: Group 1 (*p* < 0.001), Group 2 (*p* < 0.001), and Group 3 (*p* = 0.008).

#### 3.1.5. The Mental Health of the Short Form-12 Health Survey

**Figure 5:**
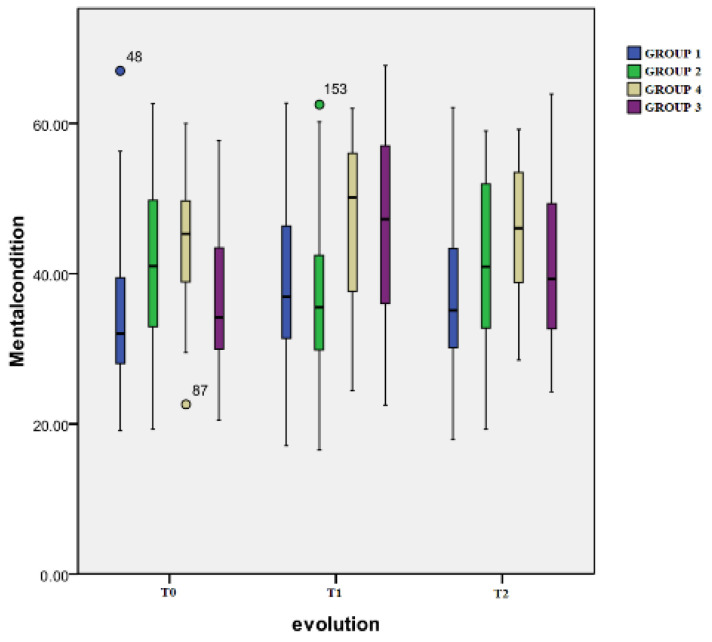
Mental condition scale.

The results obtained by computing the scores from the Mental Health of the Short Form-12 health survey are presented in Figure 5.

Time evolution: Group 4 showed significant differences in the scores at the T0 (mean: 44.2 ± 13.2) and T2 evaluations (50.1 ± 12,7) (*p* = 0.002). Group 3 displayed a highly significant increase in the scores between T0 (34.1 ± 13.4) and T1 (mean: 51.7 ± 12.2) (*p* < 0.001); this was also observed between T0 and T2 (39.1 ± 13.4) (*p* < 0.001). For Group 1 and Group 2, no significant changes were registered.

Inter-group differences: At T1 evaluation, notable differences were found between these pairs: Group 4 and Group 1 (*p* = 0.006), Group 4 and Group 2 (*p* < 0.001), Group 3 and Group 1 (*p* = 0.02), and Group 3 and Group 2 (*p* = 0.002). At T2, the only significant difference registered was between Group 1 and Group 4 (*p* = 0.014).

#### 3.1.6. Medication Intake

**Figure 6:**
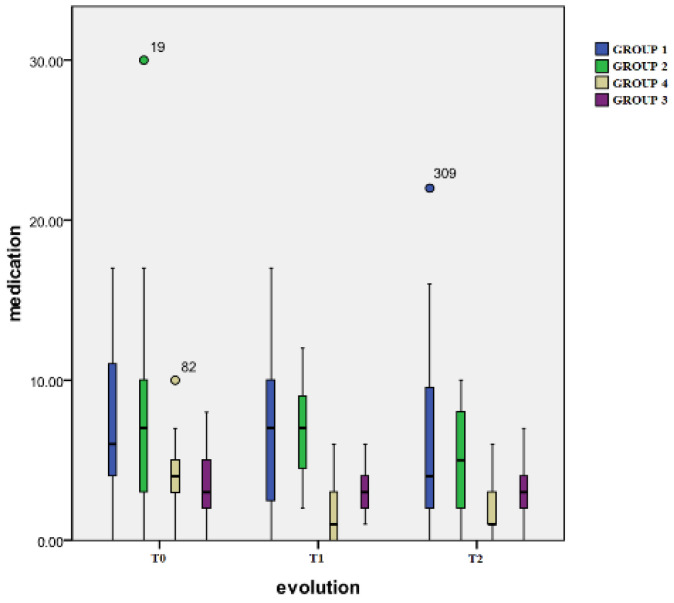
Medication intake.

The variation in the weekly medication intake of all four groups is represented in Table 3 and Figure 6.

Time evolution: For Group 2, Group 1, and Group 3, no significant differences were found regarding the medication intake between the three evaluation moments. Group 4 displayed significant differences between T0 and T1 (*p* < 0.001) and T1 and T2 (*p* = 0.005) as well as T0 and T2 (*p* < 0.001).

Inter-group differences: At the T1 moment, significant differences were still noted between the same pairs: samples involving Group 4 and Group 2 (*p* < 0.001), Group 4 and Group 1 (*p* < 0.001), samples implicated in Group 3 and Group 1 (*p* = 0.004), and Group 3 and Group 2 (*p* < 0.001). At T2, significant differences were registered between Group 4 and Group 1 (*p* = 0.02), between Group 3 and Group 1 (*p* = 0.02), and between Group 3 and Group 2 (*p* = 0.018).

### 3.2. Parameter Correlations

#### 3.2.1. Functional Impairment

**Figure 7:**
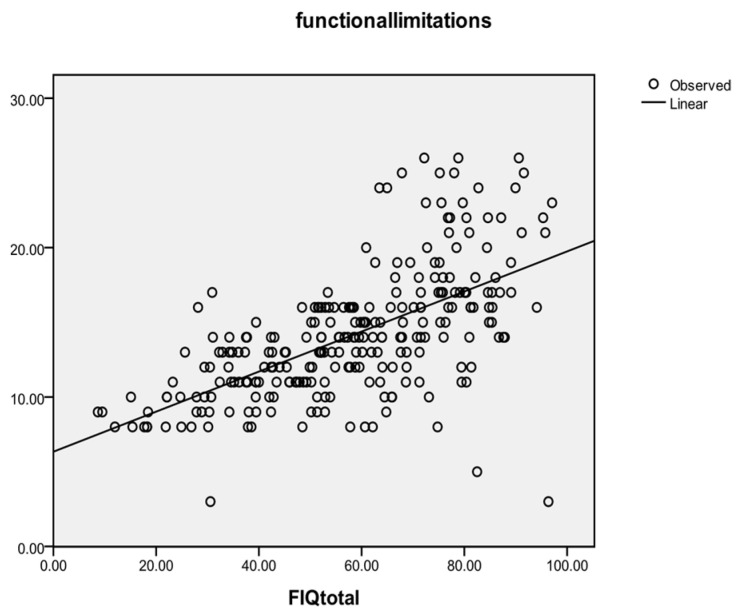
Correlation between observed functional limitation and total FIQ.

A direct correlation (total r = 0.478, total *p* < 0.001; for Group 3: r = 0.564, for Group 4: r = 0.446, for Group 1: r = −0.031) was found between the observed impairment (represented by the observed functional limitations) and the impairment as perceived by the patients (represented by the scores obtained from the first item of FIQ: FIQ1). Reverse general correlations were found between the observed functional limitations and the physical scale of the SF-12 (r = −0.518, *p* < 0.001) or the mental scale of the SF-12 (r = −0.489). The observed functional limitations score also correlates with the FIQ total score at a general level (r = 0.615, *p* < 0.001; Pre r = 0.430, Post r = 0.657, Follow-up r = 0.709), as demonstrated in Figure 7.

The strongest and most significant correlation registered was for Group 3 (r = 0.676, *p* < 0.001), followed by Group 4 (r = 0.592, *p* < 0.001), while Group 1 registered the weakest correlation (r = 0.307, *p* = 0.014).

#### 3.2.2. Disease Impact

**Figure 8:**
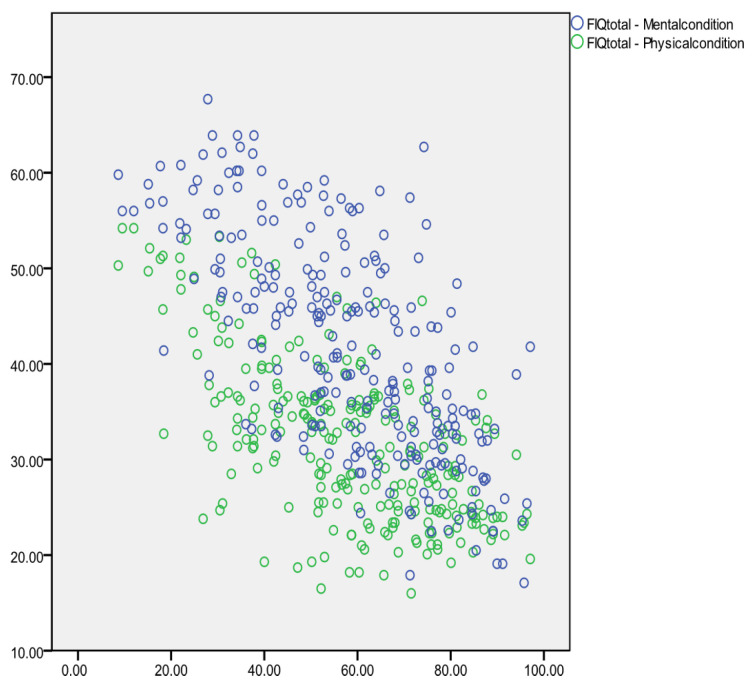
Correlation between FIQ total score and SF-12.

Reverse correlations were found between the FIQ total score and the two dimensions of the SF-12: physical condition scale (r = −0.632, *p* < 0.001; T0: r = −0.460, T1: r = −0.578, T2: r = −0.768) and mental condition scale (r = −0.696, *p* < 0.001; T0: r = −0.713, T1: r = −0.661, T2: r = −0.657), as shown in Figure 8.

## 4. Discussion

FM, a chronic illness characterized by generalized musculoskeletal pain and other symptoms, remains incompletely understood, yet significantly impacts patients’ quality of life, making it clinically imperative to investigate influencing factors [47]. The multifaceted symptoms of the disease, beyond pain alone, contribute uniquely to its spectrum [48], with neuropsychological factors notably shaping symptom perception and disease progression, distinguishing it from other rheumatologic conditions [49]. Our study aimed to explore these connections further, focusing on how educational levels influence fibromyalgia’s impact on functional areas.

Research has shown that education level has a greater influence on health behaviors than an individual’s material circumstances [50]. Additionally, certain lifestyle factors are associated with chronic musculoskeletal pain conditions, including fibromyalgia [51]. Despite a prevalence skewed towards women and those with lower education and socioeconomic status, recent unbiased studies reveal a nuanced demographic picture [52]. A few studies have explored how cultural and literacy differences affect FM experiences [53]. For instance, Büyükşireci et al. (2021) highlighted a correlation between lower education levels and greater functional impacts of FM, suggesting a critical link between literacy, education, and disease management [54].

The obtained results support that differences in the perception of the impact of the disease (using FIQ total score) were evident between the groups from the initial evaluation. This parameter was significantly influenced by the application of non-pharmacological therapy in patients with higher educational levels (at least college education). The differences were highly statistically significant when comparing patients with university degrees to those with at most a secondary education level. Research shows that higher literacy and education levels are linked to better health outcomes. Patients who comprehend their condition and treatment plans are more likely to follow prescribed therapies, heed medical advice, and engage in health-promoting behaviors. For example, the study of Berkman et al. from 2011 found that health literacy interventions significantly improved adherence to chronic disease management programs [44]. Additionally, significant differences were observed between patients with university or college education compared to those with at most a high school education, even three months after treatment cessation. The types of therapies applied have both direct and indirect effects on functional status and, consequently, on the ability to perform daily activities. The effectiveness of CBT is correlated with the patient’s understanding of their illness, as some methods, such as relaxation techniques, require a high level of patient cooperation [55]. Furthermore, according to the World Health Organization, health literacy is correlated with disability, mood disturbances, and overall health [45].

Functional limitations, assessed through both objective measures and patient-reported outcomes, were also used to evaluate the impact of illness on functioning and quality of life in FM patients. Functional limitations, evidenced through classical maneuvers, were apparent among the four groups studied from the start of the treatment. Application of kinesiotherapy and CBT significantly influenced observed functional limitations immediately after the end of non-pharmacological therapy for both groups of patients with higher education levels. At the three-month follow-up, statistical significance remained high for patients with college-level education, while for those with university education, the statistical significance diminished. These findings align with other studies that highlight the importance of patient education and physical exercise in managing FM, suggesting that the level of education may influence the perception and effectiveness of these treatments [56,57].

However, subjective evaluations of the disease impact did not consistently reflect these objective findings. A notable result in our study was that the functional limitations imposed by the disease did not vary significantly in subjective evaluations among patients, regardless of their educational level. However, a statistically significant difference was observed in the final assessments: patients with college-level education reported notably different outcomes compared to those with secondary school education or lower. Despite the correlation found between observed impairments and perceived functional limitations, subjective assessments of how the disease impacted daily activities did not reveal significant differences. In a recent systematic review, Garcia-Rios et al. concluded that patient education significantly enhances disease perception, highlighting the potential crucial role of education [58]. Additionally, in a study involving 322 Norwegian women with FM, Kurtze et al. explored various factors influencing work status. They found that perceptions of physical dysfunction due to the disease had the greatest impact on professional activity, with pain and affective elements exerting less significant influence [59]. While our results suggest that patients with higher education levels demonstrated more significant improvements in functional status, it is likely that factors beyond literacy or education play essential roles in the perception of functional limitations among this specific patient group.

Only a limited number of studies have explored the relationship between health-related quality of life and various factors associated with fibromyalgia [60]. Educational level appears to be a significant determinant of patients’ quality of life, as assessed by the SF-12 physical and mental health components, within our study cohorts. Initially, there was a noticeable difference between groups in the physical component and particularly in the mental component. Non-pharmacological therapies demonstrated significantly superior effectiveness in groups with higher educational levels. Conversely, no statistically significant improvements in well-being were observed at the end of treatment or at three months post-treatment in groups with lower educational levels. In a recent cross-sectional study, Jafari et al. [61] found that fibromyalgia adversely affects health-related quality of life. This impact is influenced by factors including body mass index (BMI), menstrual status, educational level, history of depression, and sleep quality [61]. As expected, inverse correlations were found between patients’ functional status and both components of the SF-12 scale assessing well-being. These findings are consistent with those of other studies [62,63], supporting the idea that adequate health literacy not only enhances individual health through lifestyle changes but also empowers individuals with the knowledge, skills, and confidence needed to improve public health.

Furthermore, medication consumption for pain control markedly decreased over the patient follow-up period (*p* < 0.001) among university graduates compared to all other groups. These results corroborate previous findings that underscore the high medication requirements among FM patients [64].

Although this study presents important findings, it also has certain limitations. First, the lack of research focusing on the evaluation of literacy in fibromyalgia created a notable challenge. Secondly, the limited number of subjects in each group and the inclusion of patients from only one university center may limit this study’s generalizability. Additionally, using only one test to objectively measure the degree of disability among patients may introduce bias, as the other tests relied on self-assessments. Moreover, the study focused solely on the functional status of patients, without considering other parameters such as pain or elements of daily life. Regarding internal validity, we believe that factors such as ensuring stable baseline pharmacological treatment for FM and enrolling patients who were already under the care of their treating physician at the time of the study helped minimize internal bias. Furthermore, the similar sizes of the patient cohorts, the restriction to adult participants, and the consistency in evaluations conducted by the same specialists throughout the study strengthen the internal validity of the results. The external validity of this study is supported by the fact that patients met the 2019 ACR criteria, both male and female patients were included, only those who completed at least 70% of the therapeutic program were analyzed, and the hospitals involved in the study cover a large geographic area of the country.

Implications for future research and clinical practice: currently, non-pharmacological treatments, although supported, are not uniformly evaluated or applied, and international fibromyalgia guidelines fail to incorporate personalized therapy adjustments. Recognizing individual factors, such as literacy levels, could improve patient adherence and therapy effectiveness. Future research should emphasize evaluating patient literacy in the development of therapeutic plans, particularly for non-pharmacological treatments. This perspective may need to extend beyond fibromyalgia to encompass other chronic conditions as well.

## 5. Conclusions

This study highlights the significant impact of educational level on managing fibromyalgia (FM), showing that individuals with higher education tend to have better functional outcomes and more effective disease management. Patients with higher education levels, who underwent non-pharmacological therapies such as kinesiotherapy and cognitive-behavioral therapy, saw significant improvements in their functional status and reported better overall well-being. However, despite these advancements, subjective assessments of the disease’s impact did not always match the objective improvements, suggesting that factors beyond education—like personal perceptions and external influences—also play a role. These findings emphasize the crucial role of health literacy in FM management and suggest that educational interventions may further enhance disease management and patient quality of life.

## Figures and Tables

**Table 1:** Socio-demographic characteristics of the study group.

Socio-Demographic Characteristic	Group 1 n = 32	Group 2 n = 32	Group 3 n = 30	Group 4 n = 34	*p* Value
Mean age (years ± SD)	55.4 ± 7.2	52.9 ± 8.4	54.6 ± 7.1	54.1 ± 7.9	0.620
Gender: Female (n, %)	28 (87.5)	27 (84.3)	28 (93.3)	30 (88.2)	
Residence area: Urban (n, %)	21 (65.6)	24 (75.0)	26 (86.6)	31 (91.1)	0.009
Marital status (n, %):					
Married	28 (87.5)	29 (90.6)	26 (86.6)	27 (79.4)	0.680
Divorced	1 (3.1)	2 (6.2)	3 (10.0)	4 (11.7)	<0.001
Single/Widowed	3 (9.3)	1 (3.1)	1 (3.3)	3 (8.8)	0.374
Social media access (n, %)	31 (96.8)	32 (100)	30 (100)	34 (100)	0.450
Working status (n, %):					
Employed	10 (31.2)	11 (34.3)	11 (36.6)	19 (55.8)	0.005
Unemployed	13 (40.6)	13 (40.6)	9 (30)	11 (32.3)	*p* = 0.252
Disabled	9 (28.1)	8 (25.0)	10 (33.3)	4 (11.7)	0.008
No. of cigarettes/day (mean ± SD)	4.9 ± 5.0	4.8 ± 2.1	3.3 ± 4.3	5.2 ± 4.2	0.257
Actual drug consumption (n, %)	0 (0)	0 (0)	0 (0)	0 (0)	
Alcohol consumption (n, %)	2 (3.1)	3 (9.3)	3 (10.0)	4 (11.7)	0.346
Coffee consumption (more than 1 coffee/day) (n, %)	29 (90.6)	26 (81.2)	24 (80.0)	28 (82.3)	0.125

n = number of patients; SD = standard deviation.

**Table 2:** Clinical characteristics of the study group.

Clinical Characteristic	Group 1 n = 32	Group 2 n = 32	Group 3 n = 30	Group 4 n = 34	*p* Value
Duration of illness, years (mean ± SD)	7.8 ± 7.5	7.3 ±6.9	6.6 ±7.2	6.3 ± 7.1	0.833
Duration of pain, years (mean ± SD)	8.3 ± 3.8	8.9 ±4.8	8.5 ±5.1	8.6 ± 4.1	0.960
Associated conditions (n, %):					
Irritable bowel syndrome	11 (34.3)	11 (34.3)	13 (43.3)	14 (42.05)	0.625
Chronic fatigue syndrome	9 (28.1)	11 (37.5)	11 (33.3)	12 (20.0)	0.433
Tension headaches	28 (87.5)	27 (78.1)	28 (93.3)	30 (88.2)	0.630
Observed functional limitation (mean ± SD)	12.9 ± 2.9	11.4 ± 2.8	15.4 ±2.6	13.7 ± 3.9	<0.001
FIQ1 (mean ± SD)	14.6 ± 4.8	15.1 ± 5.1	12.7 ± 4.6	12.1 ± 5.3	0.045
FIQ total (mean ± SD)	71.0 ± 15.9	74.3 ± 11.5	72.5 ± 10.5	59.3 ± 18.9	<0.001
SF12-Physical Scale (mean ± SD)	27.3 ± 12.1	26.5 ± 10.3	24.2 ± 13.3	33.0 ± 10.1	0.018
SF12-Mental Scale (mean ± SD)	32.8 ± 12.5	41.3 ± 13.2	34.1 ± 13.4	44.2 ± 13.2	0.001
Medication intake (no/week) (mean ± SD)	7.1 ± 4.9	7.7 ± 6.2	3.4 ± 2.0	3.9 ± 2.2	<0.001

FIQ = Fibromyalgia Impact Questionnaire; n = number of patients; SD = standard deviation; SF12 = Short-Form Health Survey.

**Table 3:** Medication intake.

Group	T1	T2
Group 1 (mean n ± SD)	7.2 ± 5.1	6.5 ± 6.3
Group 2 (mean n ± SD)	6.6 ± 2.9	4.9 ± 3
Group 3 (mean n ± SD)	3.3 ± 1.4	2.8 ± 1.8
Group 4 (mean n ± SD)	1.6 ± 1.8	2 ± 1.8

n = no. of tablets per week; SD = standard deviation.

## Data Availability

All data generated or analyzed during this study are included in this published article.
